# Specifying who delivers behaviour change interventions: development of an Intervention Source Ontology

**DOI:** 10.12688/wellcomeopenres.16682.1

**Published:** 2021-04-08

**Authors:** Emma Norris, Alison J. Wright, Janna Hastings, Robert West, Neil Boyt, Susan Michie

**Affiliations:** 1 Health Behaviour Change Research Group, Brunel University London, Uxbridge, UB8 3PH, UK; 2 Centre for Behaviour Change, University College London, London, WC1E 7HB, UK; 3 Research Department of Epidemiology & Public Health, University College London, London, WC1E 7HB, UK

**Keywords:** ontology, behaviour change, intervention delivery, intervention reporting, occupational groups, evidence synthesis

## Abstract

**Background:**  Identifying how behaviour change interventions are delivered, including by whom, is key to understanding intervention effectiveness. However, information about who delivers interventions is reported inconsistently in intervention evaluations, limiting communication and knowledge accumulation. This paper reports a method for consistent reporting: The Intervention Source Ontology. This forms one part of the Behaviour Change Intervention Ontology, which aims to cover all aspects of behaviour change interventions *.*

**Methods**: The Intervention Source Ontology was developed following methods for ontology development and maintenance used in the Human Behaviour-Change Project, with seven key steps: 1) define the scope of the ontology, 2) identify key entities and develop their preliminary definitions by reviewing existing classification systems (top-down) and reviewing 100 behaviour change intervention reports (bottom-up), 3) refine the ontology by piloting the preliminary ontology on 100 reports, 4) stakeholder review by 34 behavioural science and public health experts, 5) inter-rater reliability testing of annotating intervention reports using the ontology, 6) specify ontological relationships between entities and 7) disseminate and maintain the Intervention Source Ontology.

**Results:** The Intervention Source Ontology consists of 140 entities. Key areas of the ontology include *Occupational Role of Source*, *Relatedness between Person Source and the Target Population*, *Sociodemographic attributes *and *Expertise.* Inter-rater reliability was found to be 0.60 for those familiar with the ontology and 0.59 for those unfamiliar with it, levels of agreement considered ‘acceptable’.

**Conclusions:** Information about who delivers behaviour change interventions can be reliably specified using the Intervention Source Ontology. For human-delivered interventions, the ontology can be used to classify source characteristics in existing behaviour change reports and enable clearer specification of intervention sources in reporting.

## Introduction

Behaviour change interventions vary greatly in the manner they are delivered. Understanding and reporting the delivery of an intervention is essential to understanding its effectiveness, the mechanisms of change and reasons for variation in effectiveness ( Dombrowski *et al.*, 2016). To enable implementation of effective behaviour change interventions and replication of interventions in scientific enquiry, it is essential to provide clear and well-specified reporting of all aspects of interventions, including delivery ( Michie *et al.*, 2017; Michie *et al.*, 2020). The delivery of an intervention includes three core components: i) mode of delivery, i.e. how the intervention content is provided to participants such as via a website or face-to-face counselling ( Dombrowski *et al.*, 2016; Marques *et al.*, 2020), ii) schedule, i.e. how intervention content is provided to participants over the course of an intervention and iii) source, i.e. who (individually or collectively as a group or organisation) delivers the intervention content ( Michie *et al.*, 2021). In this paper, we consider intervention source.

Pre-existing relationships between an intervention’s source and participants may have an impact on intervention effectiveness. For example, peer-based interventions for health behaviours in adults may lead to greater effects than staff-delivered interventions ( Cabassa *et al.*, 2017; Webel *et al.*, 2010). Interventions are often developed to be delivered by a specific source, such as delivery of smoking cessation interventions to patients by nurses ( Rice *et al.*, 2017), and their effectiveness may not generalise to delivery by other sources ( Glasgow *et al.*, 2003). Those delivering the intervention may require certain levels of knowledge and skills, or to have undergone intervention-specific training.

Guidelines aim to improve the quality of research reporting in terms of consistency, specificity and comprehensiveness. However, the most widely used of these, the CONsolidated Standards of Reporting Trials statement (CONSORT; Schulz *et al.*, 2010) and its extension for social and psychology interventions (CONSORT-SPI; Montgomery *et al.*, 2018) do not specify reporting who delivers interventions. The Template for Intervention Description and Replication checklist (TIDieR; Hoffmann *et al.*, 2014) includes one item (Item 5: “ *Who provided – For each category of intervention provider (e.g. psychologist, nursing assistant), describe their expertise, background and any specific training given”*) but this is not further elaborated. The reporting of source characteristics within intervention reports using these brief guidelines is often poor. For example, specification of intervention providers was found to be one of four areas requiring improvement in obesity interventions ( Rauh *et al.*, 2021). We currently lack a method for specifying characteristics of an intervention’s source within behaviour change interventions. 

An appropriate method for specifying and classifying interventions is an ***ontology:*** a data structure that enables precise specification of knowledge in a given domain ( Arp *et al.*, 2015) (see glossary of italicised terms in Table 1). Ontologies provide a set of: i) unique and unambiguous identifiers representing types of ****entity**** (objects, attributes and/or processes), ii) labels and definitions corresponding to these identifiers, and ii) specified relationships between the entities ( Arp *et al.*, 2015; Larsen *et al.*, 2017; Norris *et al.*, 2019). These labels, definitions and relationships provide formal specification and a ‘controlled vocabulary’ for the given domain. Ontologies are dynamic representations that are maintained and updated according to new evidence in the given field ( Arp *et al.*, 2015; Michie & Johnston, 2017), in fields such as molecular biology ( Ashburner *et al.*, 2000). Ontologies are also machine-readable, hence suitable for ****annotation****, evidence synthesis and other computational applications ( Hastings, 2017).

**Table 1.** Glossary.

Term	Definition	Source
**Annotation**	Process of coding selected parts of documents or other resources to identify the presence of ontology entities	Michie *et al.*, 2017.
**Annotation guidance manual**	Written guidance on how to identify and tag pieces of text from intervention evaluation reports with specific codes relating to entities in the ontology, using EPPI-Reviewer software.	
**Basic Formal** **Ontology (BFO)**	An upper level ontology consisting of continuants and occurrents developed to support integration, especially of data obtained through scientific research.	Arp *et al.*, 2015.
**Continuant**	Entities within an ontology that continue to exist over time, for example, objects and spatial regions.	Arp *et al.* 2015.
**Entity**	Anything that exists, that can be a continuant or an occurrent as defined in the Basic Formal Ontology.	Arp *et al.*, 2015.
**EPPI-Reviewer**	A web-based software program for managing and analysing data in all types of systematic review (meta-analysis, framework synthesis, thematic synthesis etc. It manages references, stores PDF files and facilitates qualitative and quantitative analyses such as meta- analysis and thematic synthesis. It also has a facilitate to annotate published papers.	Thomas & Brunton, 2010. *EPPI-Reviewer 4: http://eppi.ioe.ac.uk/eppireviewer4/* *EPPI-Reviewer Web Version: https://eppi.ioe.ac.uk/eppireviewer-web/*
**GitHub**	A web-based platform used as a repository for sharing code, allowing version control.	*https://github.com/*
**Inter-rater** **reliability**	Statistical assessment of similarity and dissimilarity of coding between two or more coders. If inter-rater reliability is high this suggests that ontology entity definitions and labels are being interpreted similarly by the coders.	Gwet, 2014. *Handbook of inter-rater reliability: The definitive guide to measuring the* *extent of agreement among raters*. Gaithersburg, Advanced Analytics.
**Interoperability**	Two systems are interoperable if data coming from each system can be used by the other system. Note: An ontology is interoperable with another ontology if it can be used together with or re-uses parts from the other ontology	http://www.obofoundry.org/principles/fp-010-collaboration.html
**Issue tracker**	An online log for problems identified by users accessing and using an ontology.	BCIO Issue Tracker: https://github.com/HumanBehaviourChangeProject/ontologies/ issues
**OBO Foundry**	The Open Biological and Biomedical Ontology (OBO) Foundry is a collective of ontology developers that are committed to collaboration and adherence to shared principles. The mission of the OBO Foundry is to develop a family of interoperable ontologies that are both logically well-formed and scientifically accurate.	Smith *et al.*, 2007; *www.obofoundry.org/*
**OBO Foundry** **principles**	Good practice principles of ontology development and maintenance intended as normative for OBO Foundry ontologies. Ontologies submitted to OBO Foundry are evaluated against them.	http://www.obofoundry.org/principles/fp-000-summary.html
**Occurrent**	Entities within an ontology that extend over time, for example, processes.	Arp *et al.*, 2015.
**Ontology**	A standardised representational framework providing a set of terms for the consistent description (or “annotation” or “tagging”) of data and information across disciplinary and research community boundaries.	Arp *et al.*, 2015.
**Parent class**	A class within an ontology that is hierarchically related to one or more child (subsumed) classes such that all members of the child class are also members of the parent class and all properties of the parent class are also properties of the child class.	Arp *et al.*, 2015.
**ROBOT**	An automated command line tool for ontology workflows.	Jackson *et al.*, 2019; http://robot.obolibrary.org
**URI**	A string of characters that unambiguously identifies an ontology or an individual entity within an ontology. Having URI identifiers is one of the OBO Foundry principles.	http://www.obofoundry.org/principles/fp-003-uris.html
**Versioning**	Ontologies that have been released are expected to change over time as they are developed and refined, leading to a series of different files. Consumers of ontologies must be able to specify exactly which ontology files they used to encode their data or build their applications and be able to retrieve unaltered copies of those files in perpetuity. Versioning is one of the OBO Foundry principles.	http://www.obofoundry.org/principles/fp-004-versioning.html
**Web Ontology** **Language (OWL)**	A formal language for describing ontologies. It provides methods to model classes of “things”, how they relate to each other and the properties they have. OWL is designed to be interpreted by computer programs and is extensively used in the Semantic Web where rich knowledge about web documents and the relationships between them are represented using OWL syntax.	https://www.w3.org/TR/owl2-quick-reference/

No ontology currently exists to describe the full detail of behaviour change intervention sources ( Norris *et al.*, 2019). A comprehensive Behaviour Change Intervention Ontology (BCIO) is being developed as part of the Human Behaviour-Change Project ( Michie *et al.*, 2017; Michie *et al.*, 2020). The BCIO consists of an upper level with 42 entities, one of which is *Behaviour change intervention source*, specified as part of the Delivery in a given behaviour change intervention (BCI) scenario ( Michie *et al.*, 2021). The current study used the ontology development methodology specified for the Human-Behaviour Change Project ( Wright *et al.*, 2020) to develop a reliable ontology for specifying and classifying who delivers interventions. This paper reports the development and final version of the Intervention Source Ontology.

## Methods

The Intervention Source Ontology was developed in an iterative process of seven steps ( Wright *et al.*, 2020).

### Step 1 – Development of the scope and definition of the Intervention Source Ontology

A definition and overall topic for the ontology was set by reviewing dictionaries and the TIDieR reporting guidelines ( Hoffmann *et al.*, 2014)(March 2018).

### Step 2 – Identifying key entities and developing the preliminary Intervention Source Ontology

An initial prototype version of the ontology was developed using both a bottom-up and top-down approach. In the bottom-up approach, 100 published reports of behaviour change interventions were reviewed to develop an initial list of source characteristics. This number of reports was chosen to generate a good initial range of entities for inclusion in the ontology ( Wright *et al.*, 2020). These reports were part of a large dataset of behaviour change intervention reports partially coded for behaviour change techniques, mechanisms of action, and modes of delivery, covering a range of health behaviours ( Carey *et al.*, 2019; Michie *et al.*, 2015). 

In the top-down approach, existing classification systems related to intervention source characteristics were reviewed, including: i) occupational classification systems (e.g. International Standard Classification of Occupations (ISCO-08; Ganzeboom & Treiman, 1996; International Labour Office, 2012), ii) published ontologies containing terms related to behaviour change intervention source, via the Ontology Lookup Service and BioPortal, iii) the Patient, Intervention, Comparison, Outcome (‘PICO’) ontology developed by the Cochrane Collaboration due to its relevance for intervention trials; iv) controlled medical vocabularies (e.g. SNOMED CT, MedDRA, MeSH); and v) intervention reporting guidelines such as the Template for Intervention Description and Replication (TiDieR) ( Hoffmann *et al.*, 2014)(March – May 2018).

### Step 3 – Refining the ontology through literature annotation, discussion and revision

The preliminary ontology was revised by the research team based on annotations of published intervention evaluation reports. Using EPPI-Reviewer 4 software ( Thomas & Brunton, 2010), two researchers independently annotated 120 behaviour change intervention evaluation reports using the revised source ontology, with 80 reports on smoking cessation and 40 on physical activity interventions. This number of reports was used as no more additional entities were apparent for inclusion in the ontology ( Wright *et al.*, 2020). An open alternative to this software used for annotation is PDFAnno ( Shindo *et al.*, 2018). Information on how and when to annotate each source characteristic was provided in an ****annotation guidance manual**** to promote standardisation of the procedure. Discrepancies were discussed and the ontology structure, definitions and annotation guidance were revised accordingly. All reports were randomised controlled trials from one of three datasets: Cochrane reviews, papers annotated for behaviour change techniques and papers already annotated for Behaviour Change Techniques within the ICSmoke systematic review project ( Black *et al.*, 2020; de Bruin *et al.*, 2016)(List of papers used in development of ontology: https://osf.io/6djfk/) (June – October 2018).

### Step 4 – Expert stakeholder review

A total of 123 members of an existing international panel of behavioural scientists and public health expert stakeholders at UCL’s Centre for Behaviour Change ( Wright *et al*., 2020) were invited to give feedback on the Intervention Source Ontology resulting from Step 3. These invited experts comprised i) 90 behavioural scientists who had provided feedback on previous projects at the Centre for Behaviour Change, ii) 20 experts from under-represented countries (i.e. not USA, Australis, Canada, UK and Netherlands: the five most commonly represented countries) identified through the BCTTv1 database, and iii) 13 stakeholders who expressed interest in being involved in Human Behaviour-Change Project stakeholder initiatives in response to invitations on Twitter and the project newsletter.

Invitations to participate were sent via email, with feedback collected through an online questionnaire using Qualtrics ^TM^ software (full survey is provided as extended data ( West *et al.*, 2020)). The task was designed to take no longer than 45 minutes to complete. The task asked experts to:

1. identify the characteristics of an intervention’s source that were of *interest* to them when trying to understand variation in the effectiveness of behaviour change interventions (open-ended question). Experts were advised to consider a specific behaviour when answering this question e.g. ‘physical activity’.
2. provide feedback on the *completeness* and *comprehensiveness* of the Intervention Source Ontology.

Experts were asked to indicate: i) if there were any entities missing (if yes, which should be added), ii) if there were any entities of definitions that should be changed (if yes, what changes should be considered), and iii) if there were any entities that should be placed in a different location in the Intervention Source Ontology.

A thematic analysis of the qualitative responses was done by two researchers (EN & AW), with the larger study team providing feedback on the analysis of the results. Themes were derived directly from responses. Feedback from this expert consultation was discussed by the research team and the Intervention Source Ontology and annotation guidance were revised. Experts did not provide further feedback on these revisions (November 2018 – March 2019).

### Step 5 – Inter-rater reliability of annotations using the Intervention Source Ontology

Assessment of ****inter-rater reliability**** of the annotations by two researchers leading the development of the ontology was conducted using ****** 50 papers from Cochrane reviews (25 for smoking cessation and 25 for physical activity). This number of papers was selected as 50 papers would give a 10–15% margin of error around the estimated percentage agreement between coders ( Gwet, 2014; Wright *et al.,* 2020). Inter-rater reliability was also assessed for annotations by two behaviour change experts unfamiliar with the ontology but with experience in annotating behaviour change intervention reports. Inter-rater reliability with experts unfamiliar with the ontology was performed in two stages: i) a random sample of 50 randomised controlled trials from a database of papers coded by Behaviour Change Techniques, with no restrictions on the outcome behaviour, with revisions to the ontology made accordingly, ii) another random sample of 30 randomised controlled trials with no restrictions on the outcome behaviour from the same database. Inter-rater reliability was assessed using Krippendorff’s Alpha ( Hayes & Krippendorrff, 2007) calculated using version 1.0.0 of the Automation Inter-Rater Reliability script developed by the HBCP ( Finnerty & Moore, 2020), incorporating the python script krippendorff 0.3.2 (April 2019 – January 2021).

### Step 6 – Specifying relationships between Intervention Source Ontology entities

The research team established relationships between ontology entities to formalise the knowledge present in the ontology. This process was conducted in line with ****Basic Formal Ontology**** principles which have been used extensively in biomedical ontologies ( Arp *et al.*, 2015). The suitability of common relationships from Basic Formal Ontology ( Arp *et al.*, 2015), the Relation Ontology ( Smith *et al.*, 2005) and the Information Artifact Ontology ( Ceusters, 2012) were assessed, including the basic hierarchical relationship ‘ *is_a’* which holds between classes where one class is a subclass of another and *‘is_about’* which holds between a class representing an information-containing entity and the class that represents what the information is about (January 2021).

### Step 7 – Disseminating and maintaining the Intervention Source Ontology

The Intervention Source Ontology was initially developed as a table of entities, with separate rows for each entity annotated with a primary label, definition, synonyms, examples and relationships. When the Intervention Source Ontology was at a stable level of development for initial release, it was converted into ****Web Ontology Language (OWL)**** ( Antoniou & Van Harmelen, 2004) format, enabling it to be viewed and visualised using ontology software such as Protégé and to be compatible with other ontologies. The conversion to OWL used the ****ROBOT**** ontology toolkit library ( Jackson *et al.*, 2019), which provides a facility to create well-formatted ontologies from templates. A ROBOT template is a comma-separated values (CSV) file that can be prepared easily in common spreadsheet software, annotated with instructions for translation from spreadsheet columns to OWL language and metadata attributes. Within the input template spreadsheet, separate columns represent the entity ID (e.g. BCIO:01023), name, definition, relationship with other entities, examples and synonyms.

This OWL version of the Intervention Source Ontology was then stored on the project GitHub repository, as GitHub has an ***issue tracker*** which allows feedback to be submitted by members of the community and can be addressed in subsequent releases. The Intervention Source Ontology was also made available as part of the growing Behaviour Change Intervention Ontology within the Ontology Lookup Service ( Côté *et al.*, 2006; Jupp *et al.*, 2015)(February 2021).

## Results

### Step 1 - Defining the scope of the Intervention Source Ontology

An intervention’s source was defined as ‘ *A role played by a person, population or organisation that provides a behaviour change intervention’.* A ‘role’ in **Basic Formal Ontology (BFO)** is a type of attribute that is “externally grounded”, that is, it depends on the context or situation of an entity.

### Step 2 - Identifying key entities and developing the preliminary Intervention Source Ontology

The International Standard Classification of Occupations (- 08 version) (ISCO-08; International Labour Office, 2012) was identified as a relevant classification system for describing sources’ occupational roles. ISCO-08 is a four-level hierarchically structured classification that allows all occupations internationally to be classified into 436-unit groups. The classification gives job content (tasks and duties) priority over national education and training requirements. Therefore, occupations that involve broadly the same sets of tasks and duties are grouped in the same category in ISCO-08, even where national skill level requirements, measured in terms of formal education, are different.

In assessing the suitability of ISCO to classify occupational roles within the ontology, some minor modifications were made to comply with ontology development guidance regarding specifying entities and reducing redundancy of terms ( Grimm & Wissman, 2011). Firstly, ISCO lower-level categories representing duplicate occupations were removed to prevent repetition within the ontology. For example, *Other Clerical Support Workers* (ISCO: 44) has only one sub-level, *Other Clerical Support Workers* (ISCO: 441) and so the sub-level was removed to prevent repetition. Second, ISCO occupation categories not found to apply to intervention sources in the bottom-up review of published intervention evaluation reports were removed. Non-relevant ISCO areas were capped at the second level of the ISCO hierarchy to prevent redundant entities being included in the ontology. For example, within *Managers* (ISCO: 1), entities were capped at the second level (e.g *Chief Executives, Senior Officials and Legislators*: ISCO: 11) and did not progress to further sub-levels. Managers were not found in development work to actually deliver interventions, although they may oversee other colleagues doing so. In contrast, for health professionals, the bottom-up review of intervention reports suggested that detail represented by all four levels of the ISCO hierarchy would be relevant.

The preliminary version 0.1 ontology of the Intervention Source Ontology encompassed a 5-level hierarchical structure, containing 190 unique entities ( https://osf.io/bxqrd/). 181 of these entities were within the lower-level of *Source* representing an individual Source’s occupational role. 179 of Person source entities were from ISCO, such as *Nursing Professionals* (ISCO: 2221), *Secondary Education Teachers* (ISCO: 233) and *Psychologists* (ISCO: 2634), with the remaining two entities of *Lay Health Worker* and *Researcher not otherwise specified* added to reflect Source occupations found in intervention evaluation reports. The remaining 9 entities were within the lower levels of *Expertise of Source* including specification of the Source’s training and skills, *Source’s role dedicated to intervention* specifying whether Source delivered the intervention as part of their usual occupational role or not, and *Reimbursement or other incentives given to Source for delivering the intervention*.

### Step 3 – Refinement of the Intervention Source Ontology

Annotations of published intervention evaluations resulted in changes being made to the Source ontology.

*Entities removed from version 0.1 to version 0.2 of ontology:* Lower-levels related to occupational roles not identified as being candidate intervention sources were removed to reduce the size of the ontology, such as Travel Attendants, Conductors and Guides, Street & Market Salespersons and Other Sales Workers.

*Entities added from version 0.1 to version 0.2 of ontology:* Firstly, Person source was expanded to include *Student or trainee* and lower-levels related to this, reflecting the Source may be an individual currently studying for a qualification. Second, entities to describe *Sociodemographics of Source* were added to reflect descriptive attributes of the Source reported, such as age, gender and ethnicity. Such characteristics may be reported to characterise the Source, or may reflect selection criterion for Source, e.g recruiting sources of the same gender or from the same ethnic group as the target population. Thirdly, *Relatedness between Source and the target population* was added, with sub-levels such as *Parent or guardian*, to reflect where the Source is recruited by virtue of having a pre-existing relationship to the target population. Fourth, entities to describe *Payment model of Source* were added, with lower-levels reflecting whether the Source was rewarded in a monetary or non-monetary manner for delivering the intervention, or whether the intervention was delivered on a voluntary basis. Last, *Organisation Source* as a general higher-level term was added to reflect interventions that are delivered by an organisation rather than by a *Person Source* (individual). For example, a Stop Smoking mass media campaign in England, such as “ NHS Smokefree”, is delivered by the National Health Service (NHS) nationally ( *Organisation Source)*, whilst components are also delivered by individual staff members at local levels ( *Person Source*).

Version 0.2 of the Intervention Source Ontology as a result of these refinements had a 6-level hierarchical structure, containing 186 unique entities ( https://osf.io/7zved/).

### Step 4 – Expert stakeholder review

Of the 123 experts contacted, 103 were from ‘more-represented’ countries and 20 from ‘less-represented’ countries. Of the 34 experts that responded, 85.3% (29/34) completed the survey, with 27 from ‘well-represented’ and 7 from ‘less-represented’ countries. Experts’ responses and how these were addressed within the ontology development are reported at https://osf.io/58qkt/ ( West *et al.*, 2020).

*Entities added from version 0.2 to version 0.3 of ontology following expert stakeholder review:* First, entities to capture physical and health characteristics of Source were added, including ‘ *Source Health status*’ e.g. body mass index (BMI), body shape, appearance and ‘ *Source Target Behaviour’* e.g. Source’s smoking status or physical activity level. Second, experts requested elaboration of definition for the ISCO entity ‘ *Psychologist*’ to include a broader range of psychology areas including counselling, forensic, health and neuropsychology. Also, an entity ‘ *Source involved in co-production of intervention’* was added to reflect where those delivering the intervention were involved in its development.

Version 0.3 of the Intervention Source Ontology as a result of these expert stakeholder recommendations had a 6-level hierarchical structure, containing 196 unique entities ( https://osf.io/zfn25/).

### Step 5 – Inter-rater reliability of annotations using the Intervention Source Ontology 

Inter-rater reliability from the 50 papers annotated by the two researchers familiar with the ontology was found to be ‘acceptable’ ( *a*=0.60; https://osf.io/m3869/) ( Hayes & Krippendorrff, 2007). The random selection of 50 papers used for inter-rater reliability testing in those unfamiliar with the ontology resulted in papers with the following target behaviours: physical activity (k=19), alcohol (k=7), sexual behaviours (k=6), dietary behaviours (k=5), medication adherence (k=3) and other behaviours such as smoking, hand hygiene and screening (k=10). The inter-rater reliability for these annotations was ‘acceptable’ ( *a*=0.59; https://osf.io/swc57/). 

*Entities removed from version 0.3 to version 1 of ontology following inter-rater reliability testing:* Annotators unfamiliar with the ontology suggested that the v0.3 196-entity ontology could be shortened to improve usability. First, ISCO areas less relevant to describing Intervention Source were capped at their top-level. For example, the lower-levels of ‘ *Managers*‘ (ISCO: 1) were removed, such as ‘ *Chief Executives, Senior Officials and Legislators’* (ISCO: 11) and lower-levels of ‘ *Clerical Support Worke*r’ (ISCO: 4) were removed, such as ‘ *General and Keyboard Clerk’* (ISCO: 41), as not being involved in intervention delivery.

Annotators unfamiliar with the ontology annotated another random sample of 30 randomised controlled trials from the same database with the following target behaviours: physical activity (k=15), sexual behaviours (k=5), alcohol (k=4) and other behaviours such as diet, smoking and tooth brushing (k=6). The inter-rater reliability for these annotations was ‘acceptable’ ( *a*=0.57; https://osf.io/sg45y/). Version 1 of the Intervention Source Ontology as a result of these annotator recommendations contains 140 unique entities ( Table 2).

**Table 2.** Entity labels, definitions and examples for all Intervention Source Entities.

Name	Parent class	Definition	Examples
**BCI source** **[BCIO:010000]**	role [BFO_0000023]	A role played by a person, population or organisation that provides a BCI.	
**person [MF:0000016]**	extended organism	A member of the species Homo Sapiens.	
**person source role** **[BCIO:010001]**	BCI source	A behaviour change intervention source role that inheres in a person.	
**person source** **[BCIO:010002]**	person	A person who is the bearer of a behaviour change intervention source role.	
**personal role of** **source [BCIO:010003]**	role	A role that inheres in a person source.	
**occupational role of** **source [BCIO:010004]**	personal role of source	A personal role of source that is realised by doing a specified type of work or working in a specified way.	Interventionist, facilitator, study staff
**manager** **[BCIO:010005]**	occupational role of source	An occupational role of source that manages, plans and coordinates the overall activities of enterprises, governments and other organisations.	Chief Executive Officers; Administrative Managers; Commercial Managers
**professional** **[BCIO:010006]**	occupational role of source	An occupational role that works in knowledge building activities, applies scientific or artistic concepts and theories or teaches about the foregoing in a systematic manner.	
**science and** **engineering** **professional** **[BCIO:010007]**	professional	A professional that conducts research, improves or develops concepts, theories and operational methods or applies scientific knowledge.	Science Professional, Mathematician, Actuary, Statistician, Life Science Professional, Engineering Professional, Electrotechnology Engineer, Architect, Planner, Surveyor, Designer
**health professional** **[BCIO:010008]**	professional	A professional that improves or develops concepts, theories and operational method, and applied scientific knowledge relating to medicine, nursing, dentistry, veterinary medicine, pharmacy, and promotion of health.	Health professional; Arts therapist, Chiropractor, Dance and movement therapist, Occupational therapist, Osteopath, Podiatrist, Recreational therapist, Health staff, Clinic staff
**medical doctor** **[BCIO:010009]**	health professional	A health professional that studies, diagnoses, treats and prevents illness, disease, injury and other physical and mental impairments in humans through the application of modern medicine. They plan, supervise and evaluate the implementation of care and treatment plans by other health care providers, and conduct medical education and research activities.	District medical doctor-therapist, family medical practitioner, general practitioner, medical doctor (general), medical officer (general), physician (general), primary health care physician, resident medical officer specializing in general practice
**generalist medical** **practitioner** **[BCIO:010010]**	medical doctor	A medical doctor that diagnoses, treats and prevents illness, disease, injury and other physical and mental impairments and maintains general health in humans through application of modern medicine. They do not limit their practice to certain disease categories or methods of treatment, and may assume responsibility for the provision of continuing and comprehensive medical care to individuals, families and communities.	Primary care physician, family doctor, GP
**specialist medical** **practitioner** **[BCIO:010011]**	medical doctor	A medical doctor that diagnoses, treats and prevents illness, disease, injury and other physical and mental impairments in humans, using specialized testing, diagnostic, medical, surgical, physical and psychiatric techniques through application of the principles and procedures of modern medicine. They specialize in certain disease categories, types of patient or methods of treatment and may conduct medical education and research in their chosen areas of specialization.	Anaesthetist, Cardiologist, Emergency medicine specialist, Gynaecologist, Obstetrician, Ophthalmologist, Paediatrician, Pathologist, Preventive medicine specialist, Psychiatrist, Radiation oncologist, Radiologist, Resident medical officer in specialist training, Specialist medical practitioner (public health), Specialist physician (internal medicine), Specialist physician (nuclear medicine), Surgeon
**nursing and** **midwifery** **professional** **[BCIO:010012]**	health professional	A health professional that provides treatment and care services for people who are physically or mentally ill, disabled or infirm, and those with potential risks to health including before, during and after childbirth. They assume responsibility for the planning, management and evaluation of the care of patients, including the supervision of other health care workers, working autonomously or in teams with medical doctors and others in the practical application of preventive and curative measures.	
**nursing professional** **[BCIO:010013]**	health professional	A health professional that provides treatment, support and care services for people who are in need of nursing care due to the effects of ageing, injury, illness or other physical or mental impairment, or potential risks to health.	Clinical nurse consultant, District nurse, Nurse anaesthetist, Nurse educator, Nurse practitioner, Operating theatre nurse, Professional nurse, Public health nurse, Specialist nurse, Nursing Counsellor, Study Nurse
**midwifery** **professional** **[BCIO:010014]**	health professional	A health professional that plans, manages, provides and evaluates midwifery care services before, during and after pregnancy and childbirth. They provide delivery care for reducing health risks to women and newborn children, working autonomously or in teams with other health care providers.	Midwife
**traditional and** **complementary** **medicine professional** **[BCIO:010015]**	health professional	A health professional that examines patients, prevents and treats illness, disease, injury and other physical and mental impairments and maintains general health in humans. They do this by applying knowledge, skills and practices acquired through extensive study of the theories, beliefs and experiences originating in specific cultures.	Acupuncturist, Ayurvedic practitioner, Chinese herbal medicine practitioner, Homeopath, Naturopath, Unani practitioner
**paramedical** **practitioner** **[BCIO:010016]**	health professional	A health professional that provides advisory, diagnostic, curative and preventive medical services more limited in scope and complexity than those carried out by medical doctors. They work autonomously or with limited supervision of medical doctors, and apply advanced clinical procedures for treating and preventing diseases, injuries and other physical or mental impairments common to specific communities.	Advanced care paramedic, Clinical officer (paramedical), Feldscher, Primary care paramedic, Surgical technician
**veterinarian** **[BCIO:010017]**	health professional	A health professional that diagnoses, prevents and treats diseases, injuries and dysfunctions of animals.	Animal pathologist, Veterinarian, Veterinary epidemiologist, Veterinary intern, Veterinary surgeon
**dentist [BCIO:010018]**	health professional	A health professional that diagnoses treats and prevents diseases, injuries and abnormalities of the teeth, mouth, jaws and associated tissues by applying the principles and procedures of modern dentistry.	Dental practitioner, Dental surgeon, Dentist, Endodontist, Oral and maxillofacial surgeon, Oral pathologist, Orthodontist, Paedodontist, Periodontist, Prosthodontist, Stomatologist
**pharmacist** **[BCIO:010019]**	health professional	A health professional that stores, preserves, compounds and dispenses medicinal products and counsel on the proper use and adverse effects of drugs and medicines following prescriptions issued by medical doctors and other health professionals.	Dispensing chemist, Hospital pharmacist, Industrial pharmacist, Retail pharmacist
**environmental** **and occupational** **health and hygiene** **professional** **[BCIO:010020]**	health professional	A health professional that assesses, plans and implements programmes to recognize, monitor and control environmental factors that can potentially affect human health, to ensure safe and healthy working conditions and to prevent disease or injury caused by chemical, physical, radiological and biological agents or ergonomic factors.	Environmental health officer, Occupational health and safety adviser, Occupational hygienist, Radiation protection expert
**physiotherapist** **[BCIO:010021]**	health professional	A health professional that assesses, plans and implements rehabilitative programmes that improve or restore human motor functions, maximize movement ability, relieve pain syndromes, and treat or prevent physical challenges associated with injuries, diseases and other impairments.	Geriatric physical therapist, Manipulative therapist, Orthopaedic physical therapist, Paediatric physical therapist, Physical therapist, Physiotherapist
**dietician and** **nutritionist** **[BCIO:010022]**	health professional	A health professional that assesses, plans and implements programmes to enhance the impact of food and nutrition on human health.	Clinical dietician, Food service dietician, Nutritionist, Public health nutritionist, Sports nutritionist
**audiologist and** **speech therapist** **[BCIO:010023]**	health professional	A health professional that evaluates, manages and treats physical disorders affecting human hearing, speech, communication and swallowing.	Audiologist, Language therapist, Speech pathologist, Speech therapist
**optometrist and** **ophthalmic optician** **[BCIO:010024]**	health professional	A health professional that provides diagnosis, management and treatment services for disorders of the eyes and visual system.	Ophthalmic optician, Optometrist, Orthoptist
**teaching professional** **[BCIO:010025]**	professional	A professional that teaches the theory and practice of one or more disciplines at different educational levels.	Education Methods Specialist, Other Language Teacher, Information Technology Teacher, Private Tutor, School Counsellor, Student Advisor
**university and higher** **education teacher** **[BCIO:010026]**	teaching professional	A teaching professional that prepares and delivers lectures and conduct tutorials in one or more subjects within a prescribed course of study at a university or other higher educational institution. They conduct research, and prepare scholarly papers and books.	Higher education lecturer, Professor, University lecturer, University tutor
**vocational education** **teacher [BCIO:010027]**	teaching professional	A teaching professional that teaches or instructs vocational or occupational subjects in adult and further education institutions and to senior students in secondary schools and colleges.	Automotive technology instructor, Cosmetology instructor, Vocational education teacher
**secondary education** **teacher [BCIO:010028]**	teaching professional	A teaching professional that teaches one or more subjects at secondary education level.	Secondary school teacher, High school teacher
**primary school** **teacher [BCIO:010029]**	teaching professional	A teaching professional that teaches teach a range of subjects at the primary education level.	
**early childhood** **educator** **[BCIO:010030]**	teaching professional	A teaching professional that promotes the social, physical, and intellectual development of children below primary school age through the provision of educational and play activities.	Early childhood educator, Pre-school teacher
**special needs teacher** **[BCIO:010031]**	teaching professional	A teaching professional that teaches children, young persons or adults with physical or intellectual special needs.	Learning disabilities special education teacher, Learning support teacher, Remedial teacher, Teacher of gifted children, Teacher of the hearing impaired, Teacher of the sight impaired
**music teacher** **[BCIO:010032]**	teaching professional	A teaching professional that teaches students in the practice, theory and performance of music in private or small group tuition or within mainstream educational institutions.	Guitar teacher (private tuition), Piano teacher (private tuition), Singing teacher (private tuition), Violin teacher (private tuition)
**arts teacher** **[BCIO:010033]**	teaching professional	A teaching professional that teaches students in the practice, theory and performance of dance, drama, visual and other arts (excluding music) in private or small group tuition or within mainstream educational institutions.	Dance teacher (private tuition), Drama teacher (private tuition), Painting teacher (private tuition), Sculpture teacher (private tuition)
**legal, social and** **cultural professional** **[BCIO:010034]**	professional	A professional that conducts research, improves or develops concepts, theories and operational methods, or applies knowledge relating to the law, social or cultural studies.	
**legal professional** **[BCIO:010035]**	legal, social and cultural professional	A legal, social and cultural professional that conducts research on legal problems, advises clients on legal aspects of problems, pleads cases or conducts prosecutions in courts of law, presides over judicial proceedings in courts of law and draft laws and regulations.	Lawyer, Judge, Coroner
**librarian, archivist** **and curator** **[BCIO:010036]**	legal, social and cultural professional	A legal, social and cultural professional that develops and maintains collections of archives, libraries, museums, art galleries and similar establishments.	Librarian, Archivist, Curator
**social professional** **[BCIO:010037]**	legal, social and cultural professional	A legal, social and cultural professional that conducts research, improves or develops concepts, theories and operational methods or applies knowledge relating to psychology, sociology, philosophy, politics, economics, sociology, anthropology, history and other social sciences, or provides social services to meet the needs of individuals and families in a community.	Economist, Sociologist, Anthropologist, Philosopher, Historian, Political Scientist
**psychologist** **[BCIO:010038]**	social professional	A social professional that studies the mental processes and behaviour of human beings as individuals or in groups, and applies this knowledge to promote personal, social, educational or occupational adjustment and development.	Clinical psychologist, Counselling psychologist, Educational psychologist, Forensic psychologist, Health psychologist, Neuropsychologist, Organizational psychologist, Psychotherapist, Sport and exercise psychologist
**social work and** **counselling** **professional** **[BCIO:010039]**	social professional	A social professional that provides advice and guidance to individuals, families, groups, communities and organizations in response to social and personal difficulties. They assist clients to develop skills and access resources and support services needed to respond to issues arising from unemployment, poverty, disability, addiction, criminal and delinquent behaviour, marital and other problems.	Counsellor, Addictions counsellor, Bereavement counsellor, Child and youth counsellor, District social welfare officer, Family counsellor, Marriage counsellor, Parole officer, Probation officer, Sexual assault counsellor, Social worker, Women’s welfare organizer
**religious professional** **[BCIO:010040]**	legal, social and cultural professional	A legal, social and cultural professional that functions as a perpetuator of sacred traditions, practices and beliefs. They conduct religious services, celebrate or administer the rites of a religious faith or denomination, provide spiritual and moral guidance and perform other functions associated with the practice of a religion.	Bonze, Imam, Minister of religion, Poojari, Priest, Rabbi
**author and journalist** **[BCIO:010041]**	legal, social and cultural professional	A legal, social and cultural professional that conceives and creates literary works, and interprets and communicates news and public affairs through the media.	Author, Writer, Journalist, Translator, News anchor
**linguist [BCIO:010042]**	legal, social and cultural professional	A legal, social and cultural professional that translates or interprets from one language into another.	Interpretator; other linguist
**creative and** **performing artist** **[BCIO:010043]**	legal, social and cultural professional	A legal, social and cultural professional that communicates ideas, impressions and facts in a wide range of media to achieve particular effects, interprets a composition such as a musical score or a script to perform or direct the performance, and hosts the presentation of such performance and other media events.	Visual artist, Musician, Singer, Composer, Dancer, Choreographer, Director, Producer, Actor, Announcer on radio, television and other media
**technician and** **associate professional** **[BCIO:010044]**	professional	A professional that performs technical and related tasks connected with research and the application of scientific or artistic concepts and operational methods, and government or business regulations.	
**health associate** **professional** **[BCIO:010045]**	technician and associate professional	A technician and associate professional that performs technical and practical tasks to support diagnosis and treatment of illness, disease, injuries and impairments in humans or animals, and supports the implementation of health care usually established by medical, veterinary, nursing and other health professionals.	Veterinary Technician
**medical and** **pharmaceutical** **technician** **[BCIO:010046]**	health associate professional	A health associate professional that performs technical tasks to assist in diagnosis and treatment of illness, disease, injuries and impairments.	Medical imaging and therapeutic equipment technician, Medical and Pathology laboratory technician, Pharmaceutical technician, Medical and dental prosthetic technician
**nursing and** **midwifery associate** **professional** **[BCIO:010047]**	health associate professional	A health associate professional that provides basic nursing and personal care for people who are physically or mentally ill, disabled or infirm, and for others in need of care due to potential risks to health including before, during and after childbirth. They generally work under the supervision of, and in support of, implementation of health care, treatment and referrals plans established by medical, nursing, midwifery and other health professionals.	Practice assistant, Quit Smoking Counsellor, Health educator, Dispensing Optician, Information Technician
**nursing associate** **professional** **[BCIO:010048]**	nursing and midwifery associate professional	A nursing and midwifery associate professional that provides basic nursing and personal care for people in need of such care due to effects of ageing, illness, injury, or other physical or mental impairment. They generally work under the supervision of, and in support of, implementation of health care, treatment and referrals plans established by medical, nursing and other health professionals.	Assistant nurse, Associate professional nurse, Enrolled nurse, Practical nurse
**midwifery associate** **professional** **[BCIO:010049]**	nursing and midwifery associate professional	A nursing and midwifery associate professional that provides basic health care and advice before, during and after pregnancy and childbirth. They implement care, treatment and referral plans usually established by medical, midwifery and other health professionals.	Assistant midwife, Traditional midwife
**traditional and** **complementary** **medicine associate** **professional** **[BCIO:010050]**	health associate professional	A health associate professional that prevents and treats human physical and mental illnesses, disorders and injuries using herbal and other therapies based on theories, beliefs and experiences originating in specific cultures. They administer treatments using traditional techniques and medicaments, either acting independently or according to therapeutic care plans established by a traditional medicine or other health professional.	Acupuncture technician, Ayurvedic technician, Bonesetter, Herbalist, Homeopathy technician, Scraping and cupping therapist, Village healer, Witch doctor
**dental assistant** **and therapist** **[BCIO:010051]**	health associate professional	A health associate professional that provides basic dental care services for the prevention and treatment of diseases and disorders of the teeth and mouth, according to care plans and procedures established by a dentist or other oral health professional.	Dental assistant, Dental hygienist, Dental therapist
**community health** **worker [BCIO:010052]**	health associate professional	A health associate professional that provides health education, referral and follow-up, case management, basic preventive health care and home visiting services to specific communities. They provide support and assistance to individuals and families in navigating the health and social services system.	Community health aide, Community health promoter, Community health worker, Village health worker, outreach worker, health worker
**physiotherapy** **technician** **and assistant** **[BCIO:010053]**	health associate professional	A health associate professional that provides physical therapeutic treatments to patients in circumstances where functional movement is threatened by injury, disease or impairment. Therapies are usually provided according to rehabilitative plans established by a physiotherapist or other health professional.	Acupressure therapist, Electrotherapist, Hydrotherapist, Massage therapist, Physical rehabilitation technician, Physiotherapy technician, Shiatsu therapist
**medical assistant** **[BCIO:010054]**	health associate professional	A health associate professional that performs basic clinical and administrative tasks to support patient care under the direct supervision of a medical practitioner or other health professional.	Clinical assistant, Medical assistant, Ophthalmic assistant
**environmental** **and occupational** **health inspector** **and associate** **[BCIO:010055]**	health associate professional	A health associate professional that investigates the implementation of rules and regulations relating to environmental factors that may affect human health, safety in the workplace, and safety of processes for the production of goods and services. They may implement and evaluate programmes to restore or improve safety and sanitary conditions under the supervision of a health professional.	Food sanitation and safety inspector, Health inspector, Occupational health and safety inspector, Pollution inspector, Product safety inspector, Sanitarian, Sanitary inspector
**ambulance worker** **[BCIO:010056]**	health associate professional	A health associate professional that provides emergency health care to patients who are injured, sick, infirm or otherwise physically or mentally impaired prior to and during transport to medical facilities.	Ambulance officer, Ambulance paramedic, Emergency medical technician, Emergency paramedic
**business and** **administration** **associate professional** **[BCIO:010057]**	technician and associate professional	A technician and associate professional that performs mostly technical tasks connected with the practical application of knowledge relating to financial accounting and transaction matters, mathematical calculations, human resource development, selling and buying financial instruments, specialized secretarial tasks and enforcing or applying government rules.	Financial and Mathematical associate professional, Sales and purchasing agent, Broker, Business Services Agent, Administrative and Specialized Secretary, Government regulatory associate professional, Medical secretary
**legal and related** **associate professional** **[BCIO:010058]**	technician and associate professional	A technician and associate professional that performs support functions in courts of law or in law offices, provide services related to such legal matters as insurance contracts, the transferring of property and the granting of loans and other financial transactions, or conduct investigations for clients.	Bailiff, Judge’s clerk, Conveyancing clerk, Court clerk, Justice of the peace, Law clerk, Legal assistant, Paralegal, Private detective, Title searcher
**social work associate** **professional** **[BCIO:010059]**	technician and associate professional	A technician and associate professional that implements social assistance programmes and community services and assist clients to deal with personal and social problems.	Community development worker, Community services worker, Crisis intervention worker, Disability services worker, Family services worker, Life skills instructor, Mental health support worker, Welfare support worker, Women’s shelter supervisor, Youth services worker
**religious associate** **professional** **[BCIO:010060]**	technician and associate professional	A technician and associate professional that provides support to ministers of religion or to a religious community, undertake religious works, preach and propagate the teachings of a particular religion and endeavour to improve well-being through the power of faith and spiritual advice.	Faith healer, Lay preacher, Monk, Nun
**sport and fitness** **worker [BCIO:010061]**	technician and associate professional	A technician and associate professional that prepares for and competes in sporting events for financial gain, trains amateur and professional sportsmen and women to enhance performance, promotes participation and standards in sport, organises and officiates sporting events, or provides instruction, training and supervision for various forms of exercise and other recreational activities.	physical activity professional, exercise physiologist, exercise therapist, exercise specialist
**athlete and sports** **player [BCIO:010062]**	sport and fitness worker	A sport and fitness worker that participates in competitive sporting events. They train and compete, either individually or as part of a team, in their chosen sport.	Athlete, Bicycle racer, Boxer, Chess player , Footballer, Golfer, Hockey player, Jockey, Poker player, Racing driver, Skier, Tennis player, Wrestler
**sports coach,** **instructor and official** **BCIO:010063]**	sport and fitness worker	A sport and fitness worker that works with amateur and professional sportspersons to enhance performance and encourage greater participation in sport, and organizes and officiates in sporting events according to established rules.	Referee, Ski instructor, Sports coach, Sports official, Swimming instructor
**fitness and recreation** **instructor and** **programme leader** **[BCIO:010064]**	sport and fitness worker	A sport and fitness worker that leads, guides and instructs groups and individuals in recreational, fitness or outdoor adventure activities.	Aerobics instructor, Fitness instructor, Horse riding instructor, Outdoor adventure guide, Personal trainer, Sailing instructor, Underwater diving instructor
**artistic, cultural and** **culinary associate** **professional** **[BCIO:010065]**	technician and associate professional	A technician and associate professional that combines creative skills and technical and cultural knowledge in creative media or food.	Photographer, Interior designers and decorator, Gallery, museum and library technician, Chef
**information and** **communications** **technician** **[BCIO:010066]**	technician and associate professional	A technician and associate professional that provides support for the day to day running of computer systems, communication systems and networks, broadcast images and sound, telecommunication signals on land, sea or in aircraft.	Information and Communications Technology Operations and User Support Technician, Telecommunications and Broadcasting Technician
**clerical support** **worker [BCIO:010067]**	occupational role of source	An occupational role that records, organizes, stores, computes and retrieves information, and performs a number of clerical duties in connection with money-handling operations, travel arrangements, requests for information, and appointments.	General and Keyboard Clerk, customers Services Clerk, Numerical and Material Recording Clerk
**services and sales** **worker [BCIO:010068]**	occupational role of source	An occupational role that provides personal or protective services related to travel, house-keeping, catering, personal care, protection against fire and unlawful acts, or sells goods in retail or markets.	
**personal services** **worker [BCIO:010069]**	services and sales worker	A services and sales worker that provides personal services related to travel, housekeeping, catering and hospitality, hairdressing and beauty treatment, animal care grooming and training, companionship and other services of a personal nature.	Travel Attendant, Conductor, Guide, Cook, Waiter, Bartender, Hairdresser, Beautician, Housekeeping Supervisor, Astrologer, Fortune-teller, Valet, Undertaker, Embalmer, Pet Groomer, Animal Care Worker
**sales worker** **[BCIO:010070]**	services and sales worker	A services and sales worker that demonstrates goods in wholesale or retail shops, at stalls and markets, door to door, via telephone or customer contact centres. They may record and accept payment for goods and services purchased, and may operate small retail outlets.	Street Salesperson, Market Salesperson, Shop Salesperson, Cashier, Ticket Clerk, Model, Sales Demonstrator, Door-to-door Salesperson, Contact Centre Salesperson, Service Station Attendant, Food Service Counter Attendant
**personal care worker** **[BCIO:010071]**	services and sales worker	A services and sales worker that provides care, supervision and assistance for children, patients and elderly, convalescent or disabled persons in institutional and residential settings.	Personal carer
**child care worker** **[BCIO:010072]**	personal care worker	A personal care worker that provides care and supervision for children in non-domestic settings.	Babysitter, Child care worker, Creche ayah, Family day care worker, Nanny, Out of school hours care worker
**teachers’ aide** **[BCIO:010073]**	personal care worker	A personal care worker that performs non-teaching duties to assist teaching staff, and provides care and supervision for children in schools and pre-schools.	Pre-school assistant, Teacher’s assistant
**health care assistant** **[BCIO:010074]**	personal care worker	A personal care worker that provides direct personal care and assistance with activities of daily living to patients and residents in a variety of health care settings, working in implementation of established care plans and practices, and under the direct supervision of medical, nursing or other health professionals or associate professionals.	Nursing aide (clinic or hospital), Patient care assistant, Psychiatric aid
**home-based** **personal care worker** **[BCIO:010075]**	personal care worker	A personal care worker that provide routine personal care and assistance with activities of daily living to persons who are in need of such care due to effects of ageing, illness, injury, or other physical or mental conditions, in private homes and other independent residential settings.	Home birth assistant, Home care aide, Nursing aide (home), Personal care provider
**protective services** **worker [BCIO:010076]**	services and sales worker	A services and sales worker that protects individuals and property against fire and other hazards, maintain law and order and enforce laws and regulations.	Firefighter, Police officer, Prison guard, Security guard, Offender manager
**skilled agricultural,** **forestry and fishery** **worker [BCIO:010077]**	occupational role of source	An occupational role that grows and harvests plants or animals to provide food, shelter and income for themselves and their households.	Market gardener, Crop grower, Animal Producer, Mixed Crop and Animal Producer, Forestry worker, Fishery worker, Hunter. Subsistence Crop Farmer, Subsistence Livestock Farmer
**craft and related** **trades worker** **[BCIO:010078]**	occupational role of source	An occupational role that applies specific technical and practical knowledge and skills to construct and maintain buildings, machinery, equipment or tools, carries out printing work, and produces or processes foodstuffs, textiles and wooden, metal and other articles, including handicraft goods.	Building Trade Worker, Metal Workers, Machinery Worker, Handicraft Worker, Printing Worker, Electrical Equipment Installers and Repairer, Electronics and Telecommunications Installer and Repairer, Food Processing Worker, Woodworker, Tobacco Product Maker
**plant and** **machine operator** **[BCIO:010079]**	occupational role of source	An occupational role that operates and monitors industrial and agricultural machinery and equipment on the spot or by remote control, or drives and operates trains, motor vehicles and mobile machinery and equipment.	Stationary Plant and Machine Operator, Driver, Mobile Plant Operator
**assembler** **[BCIO:010080]**	occupational role of source	An occupational role that assembles products from component parts according to strict specifications and procedures.	
**elementary** **occupation** **[BCIO:010081]**	occupational role of source	An occupational role that involves the performance of simple and routine tasks which may require the use of hand-held tools and considerable physical effort.	Cleaner, Labourer, Food Preparation Assistant, Street and Related Services Worker, Street Vendor (excluding Food), Refuse Worker
**armed forces** **occupation** **[BCIO:010082]**	occupational role of source	An occupational role that includes all jobs held by members of the armed forces.	Admiral, Air commodore, Air marshal, Airman, Brigadier (army), Bombardier, Captain (air force), Captain (army), Captain (navy), Colonel (army), Corporal, Field marshal, Flight lieutenant (air force), Flying officer (military), General (army), Gunner, Group captain, (air force), Lieutenant (army), Major (army), Midshipman, Naval officer (military), Navy commander, Officer cadet (armed forces), Paratrooper, Rifleman, Sergeant (army), Second lieutenant (army), Seaman, Squadron leader, Sublieutenant (navy), Wing commander
**researcher** **[BCIO:010083]**	occupational role of source	An occupational role that is a researcher but unclear what discipline.	researcher, research assistant, investigator
**student or trainee** **role [BCIO:010084]**	personal role of source	A personal role of source that is enrolled in an educational institution or a formal programme of professional training.	
**informal education** **student or trainee** **[BCIO:010085]**	student or trainee role	A student or trainee that is currently learning in a non-institutional setting.	
**school student or** **trainee [BCIO:010086]**	student or trainee role	A student or trainee that is currently learning at a primary or secondary education level in an institutional, organised setting.	
**vocational training** **student or trainee** **[BCIO:010087]**	student or trainee role	A student or trainee that is currently learning the curriculum material of vocational programme, normally in preparation for employment in a trade, job or profession.	
**higher education** **student or trainee** **[BCIO:010088]**	student or trainee role	A student or trainee that is currently learning on an advanced educational programme in a university, college or professional school.	completing a university bachelor's or master's course of study, medical school or other professional school.
**undergraduate** **student or trainee** **[BCIO:010089]**	higher education student or trainee	A higher education student or trainee that is currently studying for an undergraduate degree.	undergraduate student
**graduate student or** **trainee [BCIO:010090]**	higher education student or trainee	A higher education student or trainee that has completed an undergraduate degree.	graduate student
**masters student or** **trainee [BCIO:010091]**	higher education student or trainee	A higher education student or trainee that is currently studying for a Masters degree.	MSc student, Masters student
**doctoral student or** **trainee [BCIO:010092]**	higher education student or trainee	A higher education student or trainee that is currently studying for a doctoral degree.	PhD student
**discipline of current** **programme of** **study or training** **[BCIO:010093]**	student or trainee role	A specific domain of study undertaken by the bearer of a student or trainee role.	psychology, medicine, hairdressing
**relatedness between** **person source and** **the target population** **[BCIO:010094]**	personal role of source	A personal role of source that is realised in some relationship to the characteristics of the intervention participants.	
**family member** **[BCIO:010095]**	relatedness between person source and the target population	A relatedness between person source and the target population that is an individual who is related to another person as they are descended from a common progenitor, related by marriage or other legal tie, or by a feeling of closeness.	
**parent or guardian** **[BCIO:010096]**	family member	A family member that is a mother, father or legal carer of a child.	mother, father, step-mother, step-father, guardian
**spouse or partner** **[BCIO:010097]**	family member	A family member that is an individual who is married or in a committed relationship with another individual.	boyfriend, girlfriend, partner, fiance, wife, husband
**sibling relationship** **[BCIO:010098]**	family member	A family member that is a family relationship between two persons with at least one shared parent.	brother, sister, step-brother, step-sister
**child relationship** **[BCIO:010099]**	family member	A family member that is an offspring relationship from a person to their parent.	daughter, son, step-daughter, step-son
**carer [BCIO:010100]**	relatedness between person source and the target population	A relatedness between person source and the target population that is an individual who cares, unpaid, for a friend or family member who, due to illness or disability, requires support in their daily life activities.	carer
**friend [BCIO:010101}**	relatedness between person source and the target population	A relatedness between person source and the target population that is a person whom the participant knows, likes and trusts, typically exclusive of sexual or family relations.	
**colleague** **[BCIO:010102]**	relatedness between person source and the target population	A relatedness between person source and the target population that is a person with whom the participant works in a profession or business.	
**employer** **[BCIO:010103]**	relatedness between person source and the target population	A relatedness between person source and the target population that is a person which hires the services of the participant.	
**peer [BCIO:010104]**	relatedness between person source and the target population	A relatedness between person source and the target population that is descried as matched to intervention recipients on the basis of ‘peerness’ – age, social status, gender, shared experience, shared health status etc.	
**embedded in** **participants’** **community** **[BCIO:010105]**	relatedness between person source and the target population	A relatedness between person source and the target population that is a source who is known and working to deliver intervention in own community.	
**number of** **people delivering** **intervention to** **each participant** **[BCIO:010106]**	count data item	A count data item that is the number of providers that an individual participant in the intervention encounters.	
**total number of** **people able to** **deliver intervention** **[[BCIO:010107]**	count data item	A count data item that is the total number of providers that are available and able to deliver the intervention.	
**socio demographic** **attribute of person** **source [BCIO:010108]**	person source	A social or demographic characteristic of a human being who is the bearer of a person source role.	
**age of person source** **[BCIO:010109]**	socio demographic attribute of person source	A socio-demographic attribute of person source that is a time quality inhering in a bearer by virtue of how long the bearer has existed.	
**gender of person** **source [BCIO:010110]**	socio demographic attribute of person source	A socio-demographic attribute of person source that is an individual's perception of having a particular gender, which may or may not correspond with their birth sex.	gender
**female gender** **[BCIO:010111]**	gender of person source	A gender of person source that is the cultural gender role of female.	female, woman
**male gender** **[BCIO:010112]**	gender of person source	A gender of person source that is the cultural gender role of male.	male, man
**other gender** **[BCIO:010113]**	gender of person source	A gender of person source that reports not belonging to the cultural gender role distinctions of either male or female.	non-binary, transexual
**ethnic group** **membership of** **person source** **[BCIO:010114]**	socio demographic attribute of person source	A socio-demographic attribute of person source that is the ethnic group to which an individual identifies as belonging, where an ethic group is a population whose members have a common heritage that is real or presumed such as common culture, language, religion, behaviour or biological trait.	black, white, Indian, Chinese, Somalian
**religious group** **membership of** **person source** **[BCIO:010115]**	socio demographic attribute of person source	A socio-demographic attribute of person source that is a religious group to which an individual identifies as belonging, where a religious group is a group of people characterised by the practice of a common religion.	Muslim, Christian, Hindu, Sikh, Buddhist, Judaism
**language proficiency** **of person source** **[BCIO:010116]**	socio demographic attribute of person source	A socio-demographic attribute of person source that is an individual’s ability to speak or perform in the intervention language.	fluent, native
**health status of** **person source** **[BCIO:010117]**	socio demographic attribute of person source	A socio-demographic attribute of person source that represents their mental or physical condition.	diabetes, cancer, depression, obesity, overweight
**target behaviour** **of person source** **[BCIO:010118]**	socio demographic attribute of person source	A socio-demographic attribute of person source that is their amount or experience of the intervention’s target behaviour.	source is highly physically active
**psychological** **influence on** **intervention delivery** **of person source** **[BCIO:010119]**	socio demographic attribute of person source	A socio-demographic attribute of person source that is their existing psychological attributes related to or potentially affecting the target behaviour.	
**expertise of person** **source [BCIO:010120]**	disposition	A disposition that is an expert skill or knowledge held by the source delivering the behaviour change intervention.	
**knowledge or skill** **[BCIO:010121]**	expertise of person source	An expertise of person source that is knowledge or skills held in order to deliver the behaviour change intervention.	trained (if unclear whether pre-existing or acquired)
**pre-existing** **knowledge or skill** **[BCIO:010122]**	knowledge or skill	A knowledge or skill that is already possessed by the source which allows them to deliver the behaviour change intervention, including educational level and qualifications.	Bachelor’s degree, certification, accredited, qualified
**expertise discipline** **[[BCIO:010123]**	attribute	An attribute that is a field of knowledge or practice.	psychology, yoga, cognitive behavioural therapy, hypnotism
**discipline of pre-** **existing knowledge or** **skill [BCIO:010124]**	expertise discipline	The expertise discipline in which the person source has acquired their pre-existing knowledge and skills.	psychology, yoga, cognitive behavioural therapy, hypnotism
**acquired knowledge** **or skill [BCIO:010125]**	knowledge or skill	A knowledge or skill that is additional knowledge or skills supplied to the source to allow them to deliver the behaviour change intervention, including educational level and qualifications.	
**amount of experience** **[BCIO:010126]**	data item	A knowledge or skill that is the duration of experience in related domain held by person source.	full-day, 6 years
**affiliation to a formal** **group or organisation** **[BCIO:010127]**	pre-existing knowledge or skill	A pre-existing knowledge or skill that is recognised through affiliation to a formal group or organisation.	member of British Psychological Society
**supervision of person** **source [BCIO:010128]**	process	A process in which a person source is formally provided, by an individual with appropriate expertise, with corrective and skill- enhancing feedback, regarding the person source’s performance in delivering the intervention.	supervised
**volunteering of** **person source** **[BCIO:010129]**	process	A process in which a person source delivers the intervention on a voluntary basis without formal compensation.	voluntary basis, volunteering
**payment of person** **source [BCIO:010130]**	process	A process in which a person source is paid or compensated for delivering the intervention.	
**monetary payment** **[BCIO:010131]**	payment of person source	A payment of person source that is money, vouchers or valued objects given to the source for delivering the intervention.	paid in cash, vouchers
**non-monetary** **payment** **[BCIO:010132]**	payment of person source	A payment of person source that is non-monetary compensation given to the source for delivering the intervention.	course credit
**source role related** **to intervention** **[BCIO:010133]**	BCI source	A BCI source whose occupational or voluntary role is focused on delivery of the behaviour change intervention.	
**source involved** **in development** **of intervention** **[BCIO:010134]**	BCI source	A BCI source that is involved in the development of intervention content.	
**source involved** **in co-production** **of intervention** **[BCIO:010135]**	source involved in development of intervention	A source involved in development of intervention that has developed the intervention content in collaboration with key stakeholders such as patients or community members.	
**organisation** **[OBI:0000245]**	material entity	An entity that can bear roles, has members, and has a set of organization rules. Members of organizations are either organizations themselves or individual people. Members can bear specific organization member roles that are determined in the organization rules. The organization rules also determine how decisions are made on behalf of the organization by the organization members.	
**organisational source** **role [BCIO:010138]**	BCI source	A BCI source role that is borne by an organisation.	
**organisation source** **[BCIO:010139]**	organisation	An organisation that is the bearer of a behaviour change intervention source role, such as voluntary, public or commercial organisations delivering a behaviour change intervention.	

*Note:* BCIO = Behaviour Change Intervention Ontology; BFO = Basic Formal Ontology; MF = Mental Functioning Ontology; OBI = Ontology of Biomedical Investigations.

### Step 6 – Specifying the relationships between Intervention Source Ontology entities

Relationships from the Relation Ontology ( Smith *et al.*, 2005) were used to connect classes, namely the basic hierarchical relationship ‘ *is_a’* which holds between classes where one class is a subclass of another class (e.g *Medical doctor is_a Health professional*), ‘ *has_role’* which connects a role bearer to a role it holds (e.g *Person has_role Person source role*), and ‘ *has_participant’* where one class is involved in the process of another class (e.g *Supervision of person source has_participant Person source*). The relationship ‘ *is_about’* from the Information Artifact Ontology ( Ceusters, 2012) was also used to represent one class presenting information about another (e.g *Total number of people able to deliver intervention is_about Source*).

### Step 7 - Making the Intervention Source Ontology machine-readable and available online

A downloadable version of the final Intervention Source Ontology is available from GitHub ( Norris *et al.*, 2021). The hierarchical structure, ****URIs****, labels and definitions for all entities are described in Table 2. The ontology is accompanied by an annotation guidance manual that provides guidance on how to annotate for these entities in BCI reports (available at https://osf.io/e6dzm/).

## Discussion

This study developed the Intervention Source Ontology ( Hastings *et al.*, 2021) to specify the characteristics of who delivers behaviour change interventions, as part of the Behaviour Change Intervention Ontology ( Michie *et al.*, 2017; Michie *et al.*, 2020). The ontology consists of 140 entities across key areas of *Occupational Role of Source*, *Relatedness between Person Source and the Target Population*, *Sociodemographic attributes* and *Expertise.* Inter-rater reliability was found to be acceptable for those familiar and unfamiliar with the ontology, as assessed by Krippendorff’s alpha ( *a*=0.60 and 0.59 respectively). This suggests that the Intervention Source Ontology and associated annotating guidance can be applied with acceptable consistency, and provides confidence in the methods developed for creating the Behaviour Change Intervention Ontology ( Michie *et al.*, 2017; Wright *et al.*, 2020; https://www.humanbehaviourchange.org/).

Ontologies should be maintained and updated according to new evidence about entities and relationships ( Arp *et al.*, 2015; He *et al.*, 2018). The Intervention Source Ontology and all other ontologies within the Human Behaviour-Change Project will be updated according to advances in behavioural science and by online ontology user feedback via GitHub. When the full Behaviour Change Intervention Ontology has been released it will be submitted to the ***OBO Foundry*** ( Smith *et al.*, 2007)

### Strengths and limitations

A strength of this work is the integration of international expert stakeholder feedback in appraising and revising the ontology, a practice which is uncommon in ontology development ( Norris *et al.*, 2019). Involving a variety of domain experts provides a range of relevant knowledge and perspectives to test the ontology, a process used successfully in developing other taxonomies and frameworks (e.g. BCTTv1, Michie *et al.*, 2015; Linking BCTs and Mechanisms of Action, Carey *et al.*, 2019; MAGI framework, Borek *et al.*, 2019; TIPPME, Hollands *et al.*, 2017).

A limitation of this work is that there was a preponderance of intervention evaluation reports from high income countries, potentially limiting the ontology’s applicability to interventions in low- and middle-income countries. However, incorporating ISCO-08, an occupational classification system designed to have worldwide relevance ( International Labour Office, 2012) should enhance the Source ontology’s global applicability. A second limitation is that intervention reports annotated within the ontology development addressed only two health-related behaviours: smoking cessation and physical activity. This was due to the ontology being developed within the Human Behaviour-Change Project, which is using interventions in these behavioural domains as initial use cases ( Michie *et al.*, 2017). However, external inter-rater reliability was performed across diverse behaviours and found to be acceptable. Future application of the ontology to a wider collection of behaviours and contexts will allow it to be extended and improved.

In addition to contributing to the larger Behaviour Change Intervention Ontology, the Intervention Source Ontology provides a stand-alone classification system for describing and reporting source characteristics. It can be used to describe who, individually or organisationally, will deliver or has delivered an intervention and to synthesise evidence across studies.

## Conclusion

The Intervention Source Ontology provides a classification system that can be used reliably to specify the characteristics of who delivers interventions. It will contribute to improved research reporting and replication, simplifying the process of evidence synthesis across diverse studies. The ontology can be used in conjunction with machine-readable tools, such as the Artificial Intelligence algorithms contributing to the Knowledge System being developed within the Human Behaviour-Change Project ( Michie *et al.*, 2017). The Intervention Source Ontology is intended to act as a basis to be elaborated on in future research, as an ongoing and collaborative process. The ontology will increase understanding of what intervention sources are most effective for given intervention scenarios, varying in target population, behavioural domain, setting and a large number of intervention characteristics.

## Data availability

The data referenced by this article are under copyright with the following copyright statement: Copyright: ï¿½ 2021 Norris E et al.

Data associated with the article are available under the terms of the Creative Commons Attribution Licence, which permits unrestricted use, distribution, and reproduction in any medium, provided the original data is properly cited. 

### Underlying data

Open Science Framework: Human Behaviour-Change Project. https://doi.org/10.17605/OSF.IO/EFP4X ( West *et al.*, 2020)

This project contains the following underlying data:

- Expert feedback on Intervention Source Ontology; Raw feedback received from behavioural science and public health experts; https://osf.io/58qkt/

### Extended data

Open Science Framework: Human Behaviour-Change Project. https://doi.org/10.17605/OSF.IO/EFP4X ( West *et al.*, 2020)

This project contains the following extended data:

- Papers used in development of the Intervention Source Ontology; Papers used across stages of development of the Intervention Source Ontology, with the systematic reviews that they were identified from; https://osf.io/6djfk/
- Version 0.1 Intervention Source Ontology; Initial prototype version of Intervention Source Ontology; https://osf.io/bxqrd/
- Version 0.2 Intervention Source Ontology; Second version of Intervention Source Ontology after initial annotations; https://osf.io/7zved/
- Version 0.3 Intervention Source Ontology; After expert stakeholder feedback; https://osf.io/zfn25/
- Expert feedback survey; Full survey provided to behavioural science and public health experts in review of the Intervention Source Ontology; https://osf.io/5hjcf/
- Internal inter-rater reliability testing: https://osf.io/m3869/
- External inter-rater reliability testing; 1st round: https://osf.io/swc57/
- External inter-rater reliability testing; 2nd round: https://osf.io/sg45y/
- Coding guidelines; Manual for coding using the Intervention Source Ontology; https://osf.io/e6dzm/
- OSF page for the Human Behaviour-Change Project; Homepage for all outputs across the project; https://osf.io/h4sdy/

Zenodo: HumanBehaviourChangeProject/ontologies: HumanBehaviourChangeProject/ontologies: Upper-Level, Setting, Mode of Delivery & Source ontologies. https://zenodo.org/record/4476603#.YBLtcOj7SUk ( Hastings *et al.*, 2021)

This project contains the following extended data:

- An archived version 1 of the Intervention Source Ontology

Data are available under the terms of the Creative Commons Attribution 4.0 International license (CC-BY 4.0).

## Software availability

Source code used to calculate alpha for IRR available from: https://github.com/HumanBehaviourChangeProject/Automation-InterRater-Reliability.

Archived code at time of publication: https://doi.org/10.5281/zenodo.3833816 ( Finnerty & Moore, 2020)

License: GNU General Public License v3.0 only
