# Supplementary material for: Specifying who delivers behaviour change interventions: development of an Intervention Source Ontology
Source: Wellcome Open Res. 2021 Apr 8;6:77. [Version 1] doi: 10.12688/wellcomeopenres.16682.1 (PMC8406443; doi:10.12688/wellcomeopenres.16682.1)
Supplement: Peer review history

# Peer review: Reviewer response for version 1

**DOI:** 10.21956/wellcomeopenres.18395.r45032

**Published:** 2021-08-27

**Authors:** Nicola Metrebian

**Affiliations:** 1 Institute of Psychiatry, Psychology and Neuroscience, King's College London, London, UK

This article reports on the development of an intervention source ontology for behaviour change interventions. The authors assert that identifying how behaviour change interventions are delivered, including by whom, is key to understanding their effectiveness. However, this information is reported inconsistently. The authors have set out to develop a methodology for consistently reporting who delivers behaviour change interventions (sources) as part of the Behaviour Change Intervention Ontology.

 The authors are experts in their field and this is a well-designed and important study. Trials of behaviour change interventions need to be clearly reported to allow for interpretation of findings, replication and implementation of the intervention. This ontology will be hugely helpful. The authors use methods for ontology development and maintenance used in the Behaviour change Project. The methods have some limitations which the authors highlight in their discussion. These include, that the intervention reports used for the ontology development addressed only smoking cessation and physical activity behaviours and that there were few experts from less represented countries, thus potentially narrowing the ontology. The rationale, methods and results are set out clearly.

 I have only a few minor comments.

 Introduction

 The authors state there are 3 components to delivering an intervention, mode of delivery, schedule and source. They mention that those delivering the intervention (source) may require certain levels of knowledge and skills, or to have undergone intervention specific training but don’t elaborate. Training and on-going supervision are important for ensuring interventions are delivered effectively. The authors might consider adding a couple of sentences on the importance of training and supervision.

 Results

 The authors report on the numbers of stakeholders who responded to their request for involvement (34) and responded from less represented countries (7/34). I understand 29 of the 34 completed the survey. Can they also include the numbers from less represented countries who completed the survey? 

 I was surprised to see psychologist parent class as social professional rather than health professional. I would have expected a clinical psychologist to have a parent class of health professional. Was this as a result of having all psychology disciplines in the same group? Might these have been better classified separately? 

 It’s good to see supervision has been included in the ontology but it would be useful to have more detail around the definition ie. whether ongoing and frequency.

Is the work clearly and accurately presented and does it cite the current literature?

Yes

If applicable, is the statistical analysis and its interpretation appropriate?

Not applicable

Are all the source data underlying the results available to ensure full reproducibility?

Yes

Is the study design appropriate and is the work technically sound?

Yes

Are the conclusions drawn adequately supported by the results?

Yes

Are sufficient details of methods and analysis provided to allow replication by others?

Yes

Reviewer Expertise:

Addiction, developing and evaluating behaviour change interventions, clinical trials of behaviour change interventions.

I confirm that I have read this submission and believe that I have an appropriate level of expertise to confirm that it is of an acceptable scientific standard.

# Peer review: Reviewer response for version 1

**DOI:** 10.21956/wellcomeopenres.18395.r45034

**Published:** 2021-07-29

**Authors:** Nicole Nathan

**Affiliations:** 1 School of Medicine and Public Health, University of Newcastle, Callaghan, Australia; 2 Hunter New England Population Health, Newcastle, Australia; 3 Hunter Medical Research Institute (HMRI), Newcastle, Australia

Thank you for the opportunity to review this manuscript which sought to develop an ontology for specifying who delivers interventions. This will be an important contribution for the implementation science field as it will support trialists in their reporting of interventions which is particularly important as the field is advancing in respect to adaptation and optimisation. The authors have presented a strong rationale for the need for the ontology, used and described robust methods for this study and presented the results clearly. I really enjoyed reviewing this paper and have minimal feedback for the authors, just a few questions that they may wish to consider. Congratulations.

 When I read the title of paper I immediately thought of Justin Presseau et al work (i.e. AACTT) Framework which had built off TACT Framework. I wondered why this wasn’t made mention of in your paper and how your ontology potentially links or extends this work?

 In Step 2 the authors note that 100 published reports of behaviour change interventions were reviewed. It may not matter but I was wondering about the characteristics of these reports. It may be useful to just give a brief description of the characteristics of these studies.

 The response rate in Step 4 was quite low. Do the authors think that this could have had any impact on the findings for the thematic analysis?

 This may not be universal, but one issue that we often face in our work in community settings i.e. schools, childcare services is the employment status of the individuals (e.g. casual, temporary/permanent, part-time/fulltime) who are delivering the intervention. This has a big impact on implementation interventions that are impacted by staff turnover. I was left wondering if there was somewhere in the ontology where trialists could be encouraged to capture and report this.

Is the work clearly and accurately presented and does it cite the current literature?

Yes

If applicable, is the statistical analysis and its interpretation appropriate?

Yes

Are all the source data underlying the results available to ensure full reproducibility?

Yes

Is the study design appropriate and is the work technically sound?

Yes

Are the conclusions drawn adequately supported by the results?

Yes

Are sufficient details of methods and analysis provided to allow replication by others?

Yes

Reviewer Expertise:

Implementation Science

I confirm that I have read this submission and believe that I have an appropriate level of expertise to confirm that it is of an acceptable scientific standard.

# Peer review: Reviewer response for version 1

**DOI:** 10.21956/wellcomeopenres.18395.r44393

**Published:** 2021-06-29

**Authors:** Kim Lavoie

**Affiliations:** 1 Montreal Behavioral Medicine Centre, Centre integrée universitaire de santé et services sociaux du Nord de l'Ile de Montréal (CIUSSS-NIM), Montreal, QC, Canada; 2 University of Quebec at Montreal (UQAM), Montreal, QC, Canada

**Summary**

 This paper reported on the methods for defining an Intervention Source Ontology, which is a subset of the Behaviour Change Ontology. Identifying how behaviour change interventions are delivered, including by whom, is critical for understanding how interventions work, as well as for replication. After conducting a stakeholder review with 34 behaviour and public health experts, his study identified 140 entities and includes occupational role of the source, relatedness between person source and the target population, sociodemographic characteristics and expertise. Inter-rater reliability of annotations was acceptable at about α=0.60.

**Strengths**

 This is a timely study on an important topic – defining an ontology that describes the ‘interventionist’ in the context of behavioural interventions. This is badly needed due to the high heterogeneity of existing terms used to describe interventionists in these studies, which limits replication and knowledge transfer. This work is judged to be high impact and will be of great interest to all researchers working in this field.

 The authors clearly described the 7 steps they used to define the ontology, which were appropriate. Key strengths include engagement of relevant stakeholders across multiple phases of the work (behavioural and public health experts – with an effort to include experts from underrepresented countries), conducting inter-rater reliability testing of annotations, and providing a table with clear definitions and concrete examples that should guide future research.

**Weaknesses**

 There were few weaknesses, but the paper and its contribution could be strengthened by addressing the following points:

1. Under step 4, it was reported that experts in behaviour and public health who were members of UCL’s Center for Behaviour Change were engaged as stakeholders to conduct reviews. It would be helpful to clearly define what was considered an ‘expert’ in these areas – was this based on years of experience, relevant publications, etc? Or was any member of the CBH automatically eligible? If so, please clarify the criteria for membership.
2. Related to above, did you collect any demographic information on your experts – age, sex, years of experience, current position? You seem to have collected and reported information on their country of origin, but it would be helpful to know who the experts were to interpret their perspectives.
3. When considering expert feedback, did the research team establish a-priori any criteria for decision-making, for example, on whether to retain or move an entity, based on a certain proportion of experts making the suggestions? Or was this done at the discretion of the research team?
4. Table 2 was very clear and well-constructed. However, there are some entries that require further clarification. One regards the classification of psychologist. Based on the definition provided under ‘health professional’ – it would seem appropriate to include health psychologist here (“a professional that improves or develops concepts, theories and operational method, and applied scientiﬁc knowledge relating to medicine, nursing, dentistry, veterinary medicine, pharmacy, and promotion of health). With more than 20 years in the field, I personally don’t agree with health psychologists not being listed among the professionals in the example field.
5. Related to above, ‘psychologist’ appears as its own BCI source, but the decision to classify the very broad discipline of ‘psychologist’ as a ‘social professional’ rather than a ‘health professional’ (which may include mental health) is not clear. I am not convinced that psychologists can all be subsumed under the ‘social professional’ category - as most are actually clinicians who would be considered health professionals (mental health); and a subset of psychologists - health psychologists - either study the psychosocial and behavioural determinants of physical health and/or work as interventionists to influence physical health/disease outcomes/public health. The role of psychologist needs to be better nuanced here – perhaps the team could consider grouping those who work in areas where there are clear health or physical/mental outcomes (e.g., clinical psychologist, health psychologist, counselling, neuro, community health, sport/exercise) vs those who work in areas where there are more ‘social’ outcomes (e.g., educational, organizational, forensic, community). Psychologists may also be broadly categorized into those who are interventional (clinical, health, counselling, neuro) vs non-clinical interventional (educational, organizational) vs academic (developmental, social, etc.). Bottom line, I am not convinced the role of psychologist has been appropriately nuanced.
6. “Psychotherapist is a generic term for someone that conducts psychotherapy but is not a type of psychologist or a protected term, and a broad range of professionals may also be called psychotherapists including social workers, clergy etc. So I would remove, give it its own source definition, or add to other categories.
7. For “target behaviour of person source”, I am not sure I understand what this is – is it the extent to which the interventionist personally exhibits the target behaviour they are working on with participants? Please clarify. Idem for ‘psychological influence on intervention delivery of person source.
8. For ‘knowledge or skill’, please specify if pre-existing or acquired in preparation for the study.
9. For ‘acquired knowledge or skill’, please provide an example.
10. For ‘supervision of person source’, please provide an example of supervision (e.g., supervised through weekly meetings etc.).
11. For ‘payment of person source”, please provide an example.
12. For ‘source involved in co-production’, please clarify if co-production can only occur with patients/community members or can it also occur with HCP’s?
13. For ‘organization”, please provide examples. Idem for organization source role and organization source.
14. Minor comment: There is a typo on page 6 in the paragraph describing step 4 – should read “Australia”.

Is the work clearly and accurately presented and does it cite the current literature?

Yes

If applicable, is the statistical analysis and its interpretation appropriate?

Not applicable

Are all the source data underlying the results available to ensure full reproducibility?

Yes

Is the study design appropriate and is the work technically sound?

Yes

Are the conclusions drawn adequately supported by the results?

Yes

Are sufficient details of methods and analysis provided to allow replication by others?

Yes

Reviewer Expertise:

Health psychology, behavioural medicine, behaviour change, behavioural intervention development methods, behavioural trial methods, chronic disease prevention

I confirm that I have read this submission and believe that I have an appropriate level of expertise to confirm that it is of an acceptable scientific standard, however I have significant reservations, as outlined above.
